# Determinants of rapid weight gain during infancy: baseline results from the NOURISH randomised controlled trial

**DOI:** 10.1186/1471-2431-11-99

**Published:** 2011-11-07

**Authors:** Seema Mihrshahi, Diana Battistutta, Anthea Magarey, Lynne A Daniels

**Affiliations:** 1 School of Public Health, Queensland University of Technology, Brisbane, Australia; 2 Institute of Health and Biomedical Innovation, Queensland University of Technology, Brisbane, Australia; 3 Nutrition and Dietetics, School of Medicine, Flinders University, Adelaide, Australia

## Abstract

**Background:**

Rapid weight gain in infancy is an important predictor of obesity in later childhood. Our aim was to determine which modifiable variables are associated with rapid weight gain in early life.

**Methods:**

Subjects were healthy infants enrolled in NOURISH, a randomised, controlled trial evaluating an intervention to promote positive early feeding practices. This analysis used the birth and baseline data for NOURISH. Birthweight was collected from hospital records and infants were also weighed at baseline assessment when they were aged 4-7 months and before randomisation. Infant feeding practices and demographic variables were collected from the mother using a self administered questionnaire. Rapid weight gain was defined as an increase in weight-for-age Z-score (using WHO standards) above 0.67 SD from birth to baseline assessment, which is interpreted clinically as crossing centile lines on a growth chart. Variables associated with rapid weight gain were evaluated using a multivariable logistic regression model.

**Results:**

Complete data were available for 612 infants (88% of the total sample recruited) with a mean (SD) age of 4.3 (1.0) months at baseline assessment. After adjusting for mother's age, smoking in pregnancy, BMI, and education and infant birthweight, age, gender and introduction of solid foods, the only two modifiable factors associated with rapid weight gain to attain statistical significance were formula feeding [OR = 1.72 (95%CI 1.01-2.94), P = 0.047] and feeding on schedule [OR = 2.29 (95%CI 1.14-4.61), P = 0.020]. Male gender and lower birthweight were non-modifiable factors associated with rapid weight gain.

**Conclusions:**

This analysis supports the contention that there is an association between formula feeding, feeding to schedule and weight gain in the first months of life. Mechanisms may include the actual content of formula milk (e.g. higher protein intake) or differences in feeding styles, such as feeding to schedule, which increase the risk of overfeeding.

**Trial Registration:**

Australian Clinical Trials Registry ACTRN12608000056392

## Background

Rapid weight gain during infancy is one of the strongest risk factors for obesity later in childhood [1-3] and has also been associated with increased blood pressure [4] and increased risk of diabetes [5]. Monitoring patterns of growth during infancy may be important for predicting the risk of both childhood and adult obesity [6-8]. It is well established that birthweight is associated with weight gain during infancy. Other factors which influence growth in infancy in addition to genetic factors include nutrition in infancy, maternal pre-pregnancy BMI and gestational weight gain and smoking during and after pregnancy [1,2,9].

Weight gain in infancy is closely linked with feeding practices. Formula-fed infants reach a higher weight-for-age and length-for-age Z-score by 6 months relative to breastfed infants, and this difference continues until one year of age [10,11]. A large cohort study of over 17,000 infants nested within a randomised controlled trial conducted in Belarus, was able to show a clear dose response relationship between formula feeding and increased length and weight gain,where the relationship was strongest when infants were aged from 3 to 6 months [12]. There is also evidence that shorter duration of breastfeeding is associated with higher childhood BMI [13-16], however in some studies this has not been the case [17]. In studies where a positive association was not found there was usually no effect rather than an inverse effect and this may because the studies lacked statistical power to detect an association [17]. The differences in patterns of growth may be due to the actual content of breastmilk and formula which may relate to metabolic programming and/or other factors such as self regulation of energy intake [18].

It is plausible that differences in feeding behaviours and mother-child interactions between breastfed and formula-fed infants may also be important factors influencing weight gain. Formula-fed infants have, on average, a different feeding pattern from breastfed infants, with a higher volume (total daily volume and per feed), lower frequency of feeds, and longer time interval between feeds [19,20]. In a prospective study of healthy infants, formula-fed infants had a 20-30% higher feeding volume (measured using ingested volumes) at 6 weeks than did breastfed infants, and they had fewer overall feeds at 4 months of age [19]. In another prospective study, infants who were bottlefed from birth were twice as likely to empty the bottle or cup in late infancy, according to maternal report, than infants fed breastmilk exclusively from the breast in early infancy [21]. These findings may reflect the fact that mothers who are formula feeding tend to monitor their infants' intake and are more likely to feed to schedule rather than on demand [22]. These differences in feeding behaviours suggest that mothers who formula feed may be less responsive to infant cues of hunger and satiety; hence, infants who are bottlefed may be less able to self regulate their intake compared with breastfed infants. Once established, these behaviours may be difficult to modify. This in turn may have implications for the development of healthy eating patterns in later childhood and the prevention of childhood and adult obesity [23,24].

Developing a standard definition of overweight and obesity in children in order to determine prevalence and establish trends has always been problematic [25]. In 2006 the World Health Organisation revised its growth standards for children [26,27] and defined cut off points for defining overweight and obesity in children. The WHO Child Growth Standards are widely recognised as the optimal growth charts for use regardless of ethnicity, socioeconomic status and type of feeding. Using the WHO standard curves, overweight and obesity are defined as weight-for-height >2 and >3 SDs respectively, above the World Health Organization growth standard median. Being 'at risk of overweight' was defined as a value >1 SD and ≤2 SDs above the median weight-for-height Z-score. A systematic review of rapid weight gain in infancy and subsequent obesity defined clinically relevant rapid weight gain as a difference of >0.67 SD in weight-for-age Z-score between birth and follow up [3].

Given the suggestion that interventions aimed at modifying early weight gain could prevent adult obesity [1], our aim was to determine which modifiable risk factors, especially those related to feeding practices or behaviours, are associated with rapid weight gain in early infancy. To do this we used birth data and baseline assessment information from the NOURISH early feeding trial [28].

## Methods

### Study design and participants

NOURISH is a randomised, controlled trial designed to test the effects of an intervention aimed at promoting positive feeding practices and healthy food preferences and intakes in infancy and early childhood. The study protocol and recruitment strategy for the study have been described in detail previously [28] and are outlined briefly here. The analyses presented here made use of data collected soon after birth and at the baseline assessment conducted on the total cohort just prior to randomisation and implementation of the intervention. The results of the intervention study will be available in late 2011.

A consecutive sample of first-time mothers delivering healthy infants were first approached on the postnatal wards in one of seven major hospitals in Brisbane and Adelaide, Australia from February 2008 to March 2009. Mothers were given a brief verbal and written overview of the study and invited to give written consent and details for a second contact regarding consent for full enrolment when the infants were 4-7 months old. Mother-infant pairs were eligible for inclusion if the infants were healthy, had a gestational age >35 weeks and with a birthweight above 2500 g. Only first time mothers who were at least 18 years of age, willing and able to attend assessment and educational sessions at designated metropolitan child health clinics, and who had facility with written and spoken English were invited to participate. Mother-infant pairs were excluded if the infant had any diagnosed congenital abnormality or chronic condition likely to influence normal development (including feeding behaviour) or if the mother has a documented history of domestic violence or intravenous substance use or self-reported eating, psychiatric disorders or mental health problems.

### Data collection

At the first postnatal contact in hospital, demographic data were collected by questionnaire from women who verbally consented to be in the study. Demographic data was also collected from a sample of women who did not consent to be in the study but agreed to give information on variables such as age and education status. Birthweight data were taken from hospital records at this time. Consenting mothers were contacted for the second time by mail and sent the participant information sheet, a consent form and a questionnaire. Those declining consent at the second contact were asked to complete a brief questionnaire to supplement stage one recruitment data in order to assess potential selection bias. For the women who did consent the full questionnaire containing data on main exposure variables was returned at the baseline assessment when the infant was aged 4-7 months. Some of the questions were adapted from those used in the Longitudinal Study of Australian Children [29]. At this assessment, infant weights and lengths were measured using standard procedures [30] by trained assessors. Infants were weighed naked with a digital baby scale (Model BD-585, Tanita Corporation, Tokyo, Japan) and length was determined using a measuring board (Infantometer, Model 416, SECA, Hamburg, Germany). Age- and gender-specific Z-scores (weight-for-age, length-for-age and weight-for-length) were calculated using WHO Standards which define Z-scores as a measure of standard deviations of the distance from the median value, adjusted for gender and age [26].

### Exposure variables

#### Feeding type

This data was attained using a self administered questionnaire which was collected at the time of the assessment. Feeding type was divided into the following categories: *breastfeeding exclusively *(breastmilk only with no other food or fluids), *breastfeeding fully *(breastmilk only with occasional water or juices), *combination feeding *(breast and formula feeding), and *formula feeding only*. For the purposes of this analysis, participants who fed any breastmilk were combined to compare against those infants who were fed formula only. This enabled examination of formula feeding as a risk factor for rapid weight gain. Early solid feeding (< 4months) was also a separate variable in the analysis.

#### Feeding styles

On the same questionnaire, feeding styles were assessed using two questions from the Infant Feeding Practices Questionnaire [31]. 'Do you let your baby feed whenever s/he wants to?' and 'Do you only allow your baby to feed at set times?'. Responses were recorded on a scale using 'never', 'rarely' 'sometimes' 'often' and 'always'. Mothers who responded 'never' and 'rarely' to the first question and 'often' and 'always' to the next were recoded as 'feeding to schedule' and those who responded 'often' and 'always' to the first and 'never' and 'rarely' to the second were coded as 'feeding on demand'. Those who responded otherwise were recoded as having a 'mixed' feeding style. For the purposes of this analysis participants who fed on demand and mixed feeding style were combined to compare against those infants who were feeding to schedule. This enabled examination of feeding to schedule as a risk factor for weight gain, consistent with a similar position for formula feeding as defined above.

### Outcome variables

The main outcome variable was rapid weight gain in infancy. This was defined as a greater than 0.67 change in weight-for-age Z-score from birth to assessment. This has been suggested in other studies [3,32], and may be interpreted as crossing centile lines on a growth chart.

### Statistical analyses

Demographic characteristics between infants who were used for this analysis and those who were excluded because of missing data (i.e. on either the outcome or exposure variables or any possible confounding variables) were compared to determine whether there were substantial differences. Variables associated statistically with rapid weight gain were evaluated using a multivariable logistic regression model which included the following variables: maternal age, education, BMI, and smoking during pregnancy, and infant birthweight, age and gender. Maternal age, BMI and infant age were entered into the model as continuous variables. We also tested the interaction of feeding to schedule and formula feeding because this has been reported in another recent study [33]. Data were analysed using SPSS version 18 (SPSS Inc, Chicago IL, USA). Results were expressed as the odds of rapid weight gain and associated 95% confidence intervals in each level of categorical explanatory variables relative to the specified reference category, or per unit change in continuous variables.

### Ethics

Informed consent was obtained for all women who participated in the study. Ethical approval to conduct the study was obtained from both Universities (Queensland University Technology Human Research Ethics Committee 00171 Protocol 0700000752. and Flinders Clinical Research Ethics Committee no 52/07). The NOURISH trial has been registered with the Australian Clinical Trials Registry (ACTRN 12608000056392).

## Results

### Sample characteristics

At the first postnatal contact 2169 women agreed to subsequent contact for enrolment in the trial and provided relevant details. Subsequently we were unable to contact 511, 74 became ineligible, 885 declined consent and the remaining 698 provided signed consent and underwent baseline assessment followed by randomisation. Complete data from a total of 612 mother-infant pairs were available for analysis which represented 88% of total recruited. The remaining 86 participants had missing data on demographic variables or one of the important covariates and so were not included in the analysis. A total of 18 mothers did not return a questionnaire at all. The main demographic characteristics of the population in the study and those with missing data are shown in Table 1. The ages of the infants at assessment ranged from 4.3 months to 7.3 months. Mothers who were excluded from this analysis were younger (P = 0.002) and had lower educational attainment (P = 0.035) than those in the study. There were also a greater proportion of women who were obese in the excluded group.

**Table 1:** Demographic characteristics of the study sample (N = 698)

Characteristic		Included in final analysis (n = 612)	Excluded because of missing data (n = 86)
Maternal			
	
	Mean age (years ± SD)	30.3 ± 5.2	28.6 ± 5.7
	
	Education, n (%)		
	
	Tertiary	365 (59.6%)	41 (47.7%)
	
	Trade or Technical college	141 (23.0%)	19 (22.1%)
	
	Secondary	106 (17.3%)	26 (30.2%)
	
	Income^π^		
	
	0 ≤ $70,000	276 (46.1%)	35 (52.2%)
	
	> $70,000	323 (53.9%)	32 (47.8%)
	
	BMI kg/m^2^, n (%)		
	
	Underweight (<18.5)	14 (2.3%)	0 (%)
	
	Normal weight (18.5-24.9)	299 (49.1%)	36 (44.4%)
	
	Overweight (25.0-29.9)	193 (31.7%)	24 (29.6%)
	
	Obese (≥30)	103 (16.9%)	21 (25.9%)
	
	Maternal smoking during pregnancy, n (%)		
	
	Yes	71 (11.6%)	14 (16.7%)
	
	No	541 (88.4%)	70 (83.3%)

Infant			
	
	Mean birthweight (kg ± SD)	3.5 ± 0.4	3.5 ± 0.4
	
	Mean age at assessment (months ± SD)	4.3 ± 1.0	4.5 ± 1.0
	
	Gender		
	
	Male	305 (49.8%)	39 (45.3%)
	
	Female	307 (50.2%)	54 7%)

π for income level data sample size was reduced to 599 in included and 67 in excluded group because of missing data (non-response)

### Feeding practices and styles

A total of 304 (49.7%) infants were exclusively breastfed, 46 (7.5%) were fully breastfed, 162 (26.5%) were formula fed only, and 100 (16.3%) were fed a combination of breastmilk and formula. A total of 32.5% of infants had already started on solids by the time of the assessment, and of these infants, 24% had been introduced to solids before 4 months of age. Women without a tertiary education were more likely to formula feed [OR = 1.68 (95%CI 1.18-2.51), P = 0.013] and have introduced solids early (<4mo)[OR = 3.28 (95%CI 1.41-7.65), P = 0.007] [adjusted for age of the child, gender and other covariates]. Infants who were formula fed were more likely to have been introduced to solid foods early [OR = 2.54 (95%CI 1.26, 5.13), P = 0.009] and this finding was independent of the age of the infant.

With regard to feeding styles, 375 (61.3%) of mothers said they fed their infant on demand, 61 (10%) fed their infant to schedule and 176 (28.8%) had a mixed feeding style. For the purposes of this analysis the proportions of on demand and mixed feeding were combined and compared with proportion of infants who were fed to schedule. After adjusting for the main covariates the main modifiable factor associated with feeding on schedule was formula feeding [OR= 2.82 (95% CI 1.58-5.02), P = 0001]. The only other covariate associated with this feeding style was mothers BMI, with normal weight mothers more likely to feed to schedule than overweight or obese mothers [OR= 1.16 (95% CI 1.08-1.25), P < 0.001].

### Growth parameters and change in weight

Table 2 shows the mean Z-scores at birth and at baseline assessment, and mean weight gain. There were 84 (13.7%) infants who had a difference in weight-for-age Z-score above 0.67 defined in this analysis as 'rapid growers'[3]. At the baseline assessment 55 (9.0%) had a weight-for-length Z-score above 1 and 9 infants (1.5%) had a weight-for-length Z-score above 2 and therefore, according to WHO criteria, are at risk of overweight and overweight, respectively [34]. However, the mean weight-for-length Z-score was -0.28, which suggests that on average infants were thinner than the WHO standard. Infants who were breastfed (any) had a lower mean weight-for-age Z-score (±SD) [-0.13 (± 0.91)] at the assessment than formula fed infants [0.22 (±0.87)], P < 0.001]. Infants who were breastfed (any) also had a lower mean change in weight-for-age Z-score between birth and baseline assessment [mean change (±SD) = -0.50 (± 1.0)] than formula fed infants [mean change (±SD) = -0.2 (±1.0)], P = 0.001].

**Table 2:** Anthropometric parameters (mean ± SD) N = 612 (Z-scores calculated using WHO standards)[26]

Timepoint	Anthropometric measure	Mean ± SD
Birth	Weight (kg)	3.5 ± 0.4

	Weight Z-score	0.38 ± 0.87

Assessment	Age (months)	4.3 ± 1.0

	Weight (kg)	6.8 ± 1.0

	Length (cm)	64.1 ± 3.1

	Weight-for-age Z-score	-0.04 ± 0.92

	Length-for-age Z-score	0.33 ±0.98

	Weight-for-length Z-score	-0.28 ±1.0

	BMI-for-age Z-score	-0.31 ±0.97

	Weight gain from birth to assessment (kg)	3.3 ± 1.0

### Associations with rapid weight gain

Table 3 shows factors associated with rapid weight gain and their unadjusted and adjusted odds ratios. In the final model the main non-modifiable factors associated with rapid growth in infancy were low birthweight and gender. Lower birthweight infants put on weight more rapidly than infants who were heavier at birth and male infants were more likely to be rapid growers relative to female infants. After controlling for a number of covariates (gender, maternal age, education and smoking during pregnancy and birthweight), the only modifiable factors that showed a significant association with rapid weight gain were formula feeding [OR = 1.72 (95%CI 1.01-2.94), P = 0.047] and feeding to schedule [OR = 2.29 (95%CI 1.14-4.61), P = 0.020]. We tested the interaction of formula feeding and feeding to schedule and although they were associated, there was no interaction effect on the final model of rapid weight gain (P = 0.566).

**Table 3:** Factors associated with rapid weight gain* in infancy N = 612

Factors	OR (95% CI)	P value	AOR (95% CI)	P value
Lower birth weight vs higher birth weight	7.1 (3.60-13.7)	< 0.001	5.03 (2.82-8.99)	< 0.001

Male gender vs female	1.67 (1.04-2.67)	0.035	1.80 (1.10-2.97)	0.021

Feeding to schedule vs feeding on demand/mixed	2.52 (1.35-4.71)	0.005	2.29 (1.14-4.61)	0.020

Formula feeding only vs breastfed/combination	2.00 (1.24-3.23)	0.007	1.72 (1.01-2.94)	0.047

Early solid foods (<4mo) vs solids introduced >4mo	1.67 (0.71-4.00)	0.30	1.42 (0.54-3.71)	0.476

No smoking in pregnancy vs smoking	1.28 (0.59-2.79)	0.713	1.91(0.80-4.61)	0.148

Mother non tertiary educated vs tertiary	1.41 (0.88-2.23)	0.152	1.27 (0.75-2.15)	0.382

Model adjusted for age of child, mothers BMI and mothers age (continuous variables), AOR=Adjusted odds ratio

* change in weight-for-age Z-score from birth to assessment >0.67

We investigated the results on infant feeding type and style in more detail in order to test for a dose response effect. With regard to feeding type, although there was a higher proportion of infants classified as 'rapid growers' in the formula fed group, followed by combination fed, with the lowest proportion in the exclusively breastfed group, there was no statistically significant dose response effect (chi-squared for trend P = 0.224). Similarly for feeding style, although there was a higher proportion of 'rapid growers' in the feeding to schedule group, followed by mixed fed and a lower proportion in the feeding on demand group, there was no statistically significant dose response effect (chi-squared for trend P = 0.160).

## Discussion

In this analysis the two main modifiable factors associated with rapid weight gain in early infancy were formula feeding and feeding on schedule. Formula feeding has been well established as a likely risk factor for excessive early weight gain [35-37] however our finding that feeding style may also be related to weight gain is novel and suggests that both the content of formula milk as well as feeding dynamics, may be important for preventing rapid weight gain in infancy.

It is well known that rapid weight gain in early life is associated with later obesity and that weight gain is modified by feeding types and practices. Duration of any breastfeeding has been associated with a modest but consistent protective effect against later obesity in numerous observational studies and in three meta-analyses [16,38,39], but the mechanisms for this are still not defined clearly. The actual content of breastmilk including its high fat and low protein content, together with numerous immune related components and biologically active compounds are thought to play a major role in the protective effect [40]. It is also possible that one of the mechanisms behind the relationship between breastfeeding and obesity may be behavioural. A number of studies have shown that breastfed infants seem to self regulate their intake better than formula fed infants [21,41] and in one study in exclusively breastfed infants, their intake was inversely associated with the energy density and fat content of the breastmilk [42]. A recent review of evidence suggests that although most infants have some ability to self regulate intake in early life, not all infants are able to readjust their intake back to baseline levels after caregiver interventions [43]. This suggests that behavioural factors such as patterns of maternal control over feeding and feeding to schedule may be important mechanisms behind the relationship between breastfeeding and childhood obesity. Our findings support this hypothesis.

A recent analysis of the KOALA birth cohort study [33], which included 2834 infants in the Netherlands, showed that breastfeeding duration was inversely associated with weight gain in the first year of life and children gained on average 37.6 g less in their first year for each additional month of breastfeeding, P < 0.001. Consistent with this they found that with each additional month of breastfeeding a significantly decreased odds of being overweight at one year [OR = 0.96 (95% CI 0.93-1.00), P < 0.05)]. These findings are comparable with the results of our study. However, they also found that although breastfeeding mothers more often fed on demand, patterns of feeding (ie. feeding on demand/to schedule or mixed feeding) were unrelated to weight gain, BMI or overweight after adjustment for breastfeeding, which differs from our findings. One possible explanation for the difference is the methods used in the study in the Netherlands where they used linear regression and absolute weight gain as the outcome, whereas our study used logistic regression and looked at rapid weight gain defined by a difference in weight-for-age Z-scores of above 0.67 as has been suggested in a systematic review [3].

A prospective study of 73 infants conducted in Canada aimed to interpret growth of infants in early life comparing the WHO and CDC growth curves. They showed a difference in Z-score of 0.5 in weight-for-age by 6 months of age between infants who were breastfed only (no formula) to 6 months of age (n = 25) and those who were formula fed only at 6 months (n = 28) [35]. Interestingly, the increased rate of weight gain occurred concurrently with changes in infant feeding, suggesting that a change from breastfeeding to formula was associated with an upward shift in the rate of weight gain. This was similar to our finding which showed that formula fed infants had a greater weight-for-age Z-score and a greater difference in Z-score between birth and assessment. The authors hypothesized that overfeeding and differences in nutrient intake and responses to hunger and satiation are responsible for their findings.

Our finding that formula fed infants were twice as likely to have introduced solid foods by the time of the assessment (in some cases earlier than 4 months) is similar to the finding of other studies [44]. We also found that lower maternal education may also be linked to early introduction of solids and formula feeding only which has also been shown in other studies [45,46].

Strengths of our study include prospective design and objective measures of outcomes by trained staff which reduced the likelihood of measurement bias. However, because our data on key exposures (feeding style and type) were collected at the same time as weight, this analysis is not strictly longitudinal. Other limitations were that important potential confounders such as gestational weight gain, mode of breastfeeding (ie. via a bottle, as in expressed breastmilk or via breast) and infant sleep duration [47] (which may be a confounder or an intermediary) were not measured in our study. Because of the large variation in age for the baseline assessment we adjusted for age of the infant in our analyses. With regard to measurement of rapid weight gain, some studies have suggested that change in weight-for-length Z-score between assessment and birth may give a better reflection of rapid weight change but because we did not collect birth length we could not perform these analyses. With regard to generalisability of the sample, mothers in our sample were well educated with a low rate of maternal smoking, and the majority of families had incomes greater than A$70,000 per year, indicating that our sample was from a middle class background and therefore reflecting the population characteristics of the cities of Brisbane and Adelaide [48]. Almost half of mothers in our sample were overweight/obese (48.6%); however, this may have been due to the fact that many of them had not returned to their pre-pregnancy weight status by 4-7 months after birth of their child.

## Conclusions

Although there have been many studies looking at the effect of breastfeeding on weight gain and later obesity, to date there have been few studies examining the effects of feeding styles as a risk factor for rapid weight gain and growth. Ours is one of the only studies to show that feeding on schedule is a risk factor for clinically significant excess weight gain in infancy. Our study is also one of the few to use breastfeeding as the 'norm' and referent group and formula feeding as the comparator group and thereby as a risk factor for rapid weight gain [49]. Because formula fed infants were more than twice as likely to be fed on schedule relative to breastfed infants, it is plausible that improving breastfeeding rates in early infancy may be effective in reducing rapid weight gain and thereby the burden of obesity at a population level. Because NOURISH is a prospective study, in future reports we can examine the predictive value of rapid weight gain in infancy on obesity risk in this Australian sample. Our future plans are to follow the cohort to two years of age when predictors of overweight and obesity can be determined. This study contributes to the already established literature that rapid weight gain is associated with formula feeding, however our finding that feeding to schedule is also associated with weight gain is novel. An application of our results could be that if mothers choose to start on formula they should receive anticipatory guidance to promote feeding on demand, thus allowing the infant to better regulate their own intake.

## Competing interests

We declare no competing interests. SMs Postdoctoral Fellowship was funded by HJ Heinz.

## Authors' contributions

LD is the chief investigator of NOURISH and designed and obtained funding for the study with AM and DB. SM cleaned the data, carried out the analysis and wrote the first draft. DB provided statistical advice. All authors contributed to drafts and read and approved the final manuscript.

## Pre-publication history

The pre-publication history for this paper can be accessed here:

http://www.biomedcentral.com/1471-2431/11/99/prepub
